# Gene Expression and Functional Studies of the Optic Nerve Head Astrocyte Transcriptome from Normal African Americans and Caucasian Americans Donors

**DOI:** 10.1371/journal.pone.0002847

**Published:** 2008-08-06

**Authors:** Haixi Miao, Lin Chen, Sean M. Riordan, Wenjun Li, Santiago Juarez, Andrea M. Crabb, Thomas J. Lukas, Pan Du, Simon M. Lin, Alexandria Wise, Olga A. Agapova, Ping Yang, Charles C. Gu, M. Rosario Hernandez

**Affiliations:** 1 Department of Ophthalmology, Feinberg School of Medicine, Northwestern University, Chicago, Illinois, United States of America; 2 Department of Molecular Pharmacology and Biological Chemistry, Feinberg School of Medicine, Northwestern University, Chicago, Illinois, United States of America; 3 Robert H, Lurie Comprehensive Cancer Center, Feinberg School of Medicine, Northwestern University, Chicago, Illinois, United States of America; 4 Department of Biology, City College of New York, New York, New York, United States of America; 5 Department of Ophthalmology and Visual Sciences, Washington University School of Medicine, St. Louis, Missouri, United States of America; 6 Department of Pediatrics, Washington University School of Medicine, St. Louis, Missouri, United States of America; 7 Division of Biostatistics, Washington University School of Medicine, St. Louis, Missouri, United States of America; Minnesota State University Mankato, United States of America

## Abstract

**Purpose:**

To determine whether optic nerve head (ONH) astrocytes, a key cellular component of glaucomatous neuropathy, exhibit differential gene expression in primary cultures of astrocytes from normal African American (AA) donors compared to astrocytes from normal Caucasian American (CA) donors.

**Methods:**

We used oligonucleotide Affymetrix microarray (HG U133A & HG U133A 2.0 chips) to compare gene expression levels in cultured ONH astrocytes from twelve CA and twelve AA normal age matched donor eyes. Chips were normalized with Robust Microarray Analysis (RMA) in R using Bioconductor. Significant differential gene expression levels were detected using mixed effects modeling and Statistical Analysis of Microarray (SAM). Functional analysis and Gene Ontology were used to classify differentially expressed genes. Differential gene expression was validated by quantitative real time RT-PCR. Protein levels were detected by Western blots and ELISA. Cell adhesion and migration assays tested physiological responses. Glutathione (GSH) assay detected levels of intracellular GSH.

**Results:**

Multiple analyses selected 87 genes differentially expressed between normal AA and CA (P<0.01). The most relevant genes expressed in AA were categorized by function, including: signal transduction, response to stress, ECM genes, migration and cell adhesion.

**Conclusions:**

These data show that normal astrocytes from AA and CA normal donors display distinct expression profiles that impact astrocyte functions in the ONH. Our data suggests that differences in gene expression in ONH astrocytes may be specific to the development and/or progression of glaucoma in AA.

## Introduction

Primary open angle glaucoma (POAG), the most common form of glaucoma, is a blinding disease that affects older adults [1]. POAG in many individuals is associated with elevated intraocular pressure (IOP), a common risk factor [2]. Visual impairment in glaucoma is due to progressive loss of retinal ganglion cells (RGC) that clinically presents loss of visual field and cupping of the optic disc [3]. The site of initial damage to the retinal neurons in glaucoma is thought to be at the level of the lamina cribrosa in the optic nerve head (ONH) [4].

Astrocytes, the major glial cell type in the ONH in humans, provide cellular support function to the axons while interfacing between connective tissue surfaces and surrounding blood vessels [5]. In response to elevated IOP in human POAG and in experimental glaucoma, astrocytes undergo marked phenotypic changes [5]. Changes from the quiescent to the reactive astrocyte phenotype and the onset and progression of various human central nervous system (CNS) diseases are well established [6], [7]. In POAG, reactive astrocytes express neurotoxic mediators such as nitric oxide [8] and TNF-α [9] that may damage the axons of RGCs and remodel the extracellular matrix (ECM) of the lamina cribrosa leading to loss of elasticity and resiliency and rendering the ONH more susceptible to damage [5], [10].

In this study, we referred to African American individuals (AA) as Black Americans of African ancestry and to Caucasian Americans individuals (CA) as White Americans of Western European ancestry. We used the race, gender and age identification provided with the anonymous donor history according to guidelines published in *JAMA* and *Genome Biology* [11], [12]. POAG affects AA population at least three times more often than CA population [11]. POAG is a complex, genetically heterogeneous disease. Of the three genes from 22 genetic loci that have been identified for POAG using linkage analyses, *myocilin* (*MYOC*, OMIM 601652), *optineurin* (*OPTN*, OMIM 602432), and *WD repeat-domain 36* (*WDR36*, OMIM 609669), none of these genes have been found to associate with POAG in African Americans, Afro Caribbeans or in West African populations [12], [13].

We have investigated possible differences in ONH astrocytes from populations with different genetic backgrounds, using well characterized primary human astrocytes cultures. We have found that astrocytes derived from normal AA donors exhibit differential gene expression profiles compared to astrocytes derived from normal age-matched CA donors. Our data shows that genes associated with oxidative stress, astrocyte motility, ECM structure, immune responses and the reactive astrocyte phenotype are differentially expressed in normal astrocytes from these different populations. These results demonstrate baseline differences in ONH astrocytes from human populations with different genetic backgrounds and provide a molecular framework for future analyses from normal and glaucomatous astrocytes from different populations.

## Results

We established primary cultures of ONH astrocytes from 16 normal African American donors (age 60±11) and 21 normal Caucasian American donors (age 62±12) as described in Material and Methods. For each different assay and determination, astrocytes were cultured under identical conditions until reaching 90% confluence and then processed. ONH astrocytes from the AA and CA populations displayed a uniform polygonal shape in culture and were positive for GFAP and NCAM, which are markers of ONH astrocytes *in vivo* and *in vitro* [14], [15].

### Identification of differentially expressed genes in normal AA and CA ONH astrocytes

There were no significantly different demographic variables between populations of the 24 astrocyte lines used in microarray (Table S1). We analyzed global gene expression differences in mRNA samples from primary cultures of ONH astrocytes from 12 AA normal donors (age 58±12 years) and 12 CA normal donors (age 58±11 years). We used Affymetrix GeneChip HG U133A chips for 3 AA and 3 CA donor samples [GSE9939] and HG U133A 2.0 chips for 9 AA and 9 CA donor samples [GSE9939]. Of the 22,277 gene probes in the Affymetrix GeneChips, there were 16,710 present calls, representing 10504 genes left for the analysis. Data normalized using RMA and analyzed by SAM identified a total of 132 probe sets as differentially expressed between AA and CA (P<0.01, fold change >1.3; false discovery rate was set at ≤5%). Because many of the transcripts were represented by multiple probe sets, the actual number of genes differentially expressed in AA astrocytes compared to CA astrocytes was 87. Among these, 47 genes were upregulated and 40 genes were downregulated in AA (Table S2).

**Figure 1:**
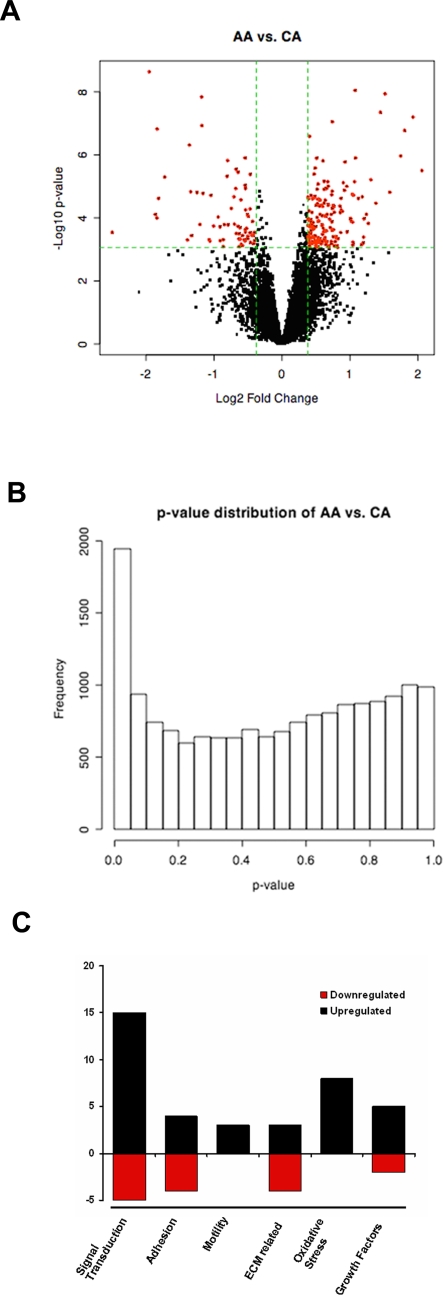
Differential gene expression in primary cultures of ONH astrocytes from age-matched normal donors (12 AA and 12 CA). A. Volcano plot indicates the size of biological effect (fold change) versus the statistical significance of the result (statistical p-value). Volcano plot represents the total number of genes used in the analysis after removing ‘absent’ genes and redundant probes (10504) on the Affymetrix Human Genome HG U133A Chip. Each point represents a gene plotted as a function of fold change (Log2 (fold change), x-axis) and statistical significance (−Log 10 (p-value), y-axis). Vertical dotted lines represent fold changes of ±1.3, respectively. The horizontal dotted line represent FDR = 0.05 (p-value is 0.00086 for this data). The red dots represent 239 selected differentially expressed genes with FDR<0.05 and fold-change >1.3. B. Estimate of the proportion of genes differentially expressed among populations. The p-value distribution of AA-CA comparison shows that a number of genes have very small p-values, which are significant even after considering the effect of multiple testing through FDR adjustment. C. Changes in gene expression in major categories in AA astrocytes, compared to CA astrocytes. The x-axis is the selected categories: signal transduction, adhesion, motility, ECM related, oxidative stress and growth factors. The y-axis is the number of genes under the category from the differentiated gene list (Table 1). Red represents the number of genes downregulated in AA and black represents number of genes upregulated in AA.

To better demonstrate the process of identifying significant genes, Figure 1A shows the volcano plot of comparison between AA and CA based on the result using the Limma package. The volcano plot indicates the size of biological effect (fold change) versus the statistical significance of the result (statistical p-value). A number of genes are shown to have high fold-change and significant p-values, which can also be seen from the p-value distribution, shown in Figure 1B. After considering the effect of multiple testing (through FDR adjustment [16]) and the size of fold-change, we identified the significant genes (with FDR<0.05 and fold-change >1.3), shown as red dots in Figure 1A. A complete list of the differential expressed genes is shown in Table S3.

**Table 1:** Selected functional categories of genes differentially expressed in AA *vs.* CA.

Gene Symbol	Gene Title	Fold Change	P-value	Chromosome location
	**Signal Tranduction**			
PSPH	phosphoserine phosphatase	2.70	0.0000	7q11.2
PDE4DIP	phosphodiesterase 4D interacting protein	2.50	0.0000	1q12
RGS5	regulator of G-protein signalling 5	2.35	0.0007	1q23.1
SOS1	son of sevenless homolog 1	1.67	0.0001	2p22-p21
RAB3B	Member RAS oncogene family	1.64	0.0006	1p32-p31
GPR56	G protein-coupled receptor 56	1.62	0.0051	16q12.2-q21
PLA2G4C	phospholipase A2, group IVC	1.60	0.0016	19q13.3
PPP1R12B	protein phosphatase 1, regulatory (inhibitor) subunit 12B, (MYPT2)	1.53	0.0026	1q32.1
MYLK	myosin, light polypeptide kinase	1.47	0.0435	3q21
CENTG2	centaurin, gamma 2	1.35	0.0103	2p24.3-p24.1
NPR3	natriuretic peptide receptor C	1.35	0.0404	5p14-p13
SYDE1	synapse defective 1, Rho GTPase, homolog 1	1.34	0.0012	19p13.12
PTK2	protein tyrosine kinase 2	1.33	0.0393	8q24-qter
ADCY3	adenylate cyclase 3	1.30	0.0300	2p23.3
ADCY9	adenylate cyclase 9	1.21	0.0216	16p13.3
TEK	TEK tyrosine kinase	−2.08	0.0006	9p21
AK3L1 (AK3)	adenylate kinase 3-like 1	−1.81	0.0055	1p31.3
STAC	SH3 and cysteine rich domain	−1.71	0.0067	3p22.3
FZD7	frizzled homolog 7	−1.45	0.0041	2q33
ADRBK2	adrenergic, beta, receptor kinase 2	−1.31	0.001	22q12.1
	**Cell Adhesion**			
WISP2	WNT1 inducible signaling pathway protein 2	2.00	0.0001	20q12-q13.1
EFNB2	ephrin-B2	1.95	0.0038	13q33
NLGN1	neuroligin 1	1.73	0.0045	3q26.31
EPB41L3	erythrocyte membrane protein band 4.1-like 3	1.66	0.0020	18p11.32
ITGA6	integrin, alpha 6	−1.64	0.0055	2q31.1
JUP	junction plakoglobin	−1.55	0.0024	17q21
ST3GAL5	ST3 beta-galactoside alpha-2,3-sialyltransferase 5	−1.50	0.0005	2p11.2
ANTXR1	anthrax toxin receptor 1	−2.30	0.0000	2p13.1
	**Cell motility**			
AMFR	autocrine motility factor receptor	2.79	0.0000	16q21
MYLK	myosin, light polypeptide kinase	1.47	0.0435	3q21
PPP1R12B	protein phosphatase 1, regulatory (inhibitor) subunit 12B	1.53	0.0026	1q32.1
	**ECM and related protein**			
ELN	elastin	2.20	0.0023	7q11.23
COL18A1	collagen type XVIII, alpha 1	1.41	0.0169	21q22.3
LTBP1	latent transforming growth factor beta binding protein 1	1.54	0.0239	2p22-p21
MFAP2	microfibrillar-associated protein 2	−1.51	0.0016	1p36.1-p35
MTCBP-1	membrane-type 1 matrix metalloproteinase cytoplasmic tail binding protein-1	−1.43	0.0012	2p25.2
NID2	nidogen 2	−1.41	0.0001	14q21-q22
PLOD2	procollagen-lysine, 2-oxoglutarate 5-dioxygenase 2	−1.60	0.0054	3q23-q24
	**Cellular detoxification/oxidative stress**			
GSTT2	glutathione S-transferase theta 2	2.82	0.0000	22q11.2; 22q11.23
GGT1	gamma-glutamyltransferase 1	1.62	0.0004	22q11.22
GGT2	gamma-glutamyltransferase 2	1.54	0.0019	22q11.1
GGTLA4	gamma-glutamyltransferase-like activity 4	1.44	0.0091	20p11.1
GSTM4	glutathione S-transferase M4	1.42	0.0245	1p13.3
GSTM1	glutathione S-transferase M1	1.38	0.0010	1p13.3
GSTM3	glutathione S-transferase M3	1.31	0.0145	1p13.3
GSTM2	glutathione S-transferase M2	1.30	0.0021	1p13.3
	**Growth factors and receptors**			
IGFBP3	insulin-like growth factor binding protein 3	1.74	0.0488	7p13-p12
FGF9	fibroblast growth factor 9 (glia-activating factor)	1.51	0.0268	13q11-q12
GFRA1	GDNF family receptor alpha 1	1.49	0.0060	10q26
CX3CL1	chemokine (C-X3-C motif) ligand 1	1.48	0.0498	16q13
IGFBP5	insulin-like growth factor binding protein 5	1.46	0.0113	2q33-q36
VEGF	vascular endothelial growth factor	−1.48	0.0051	6p12
HBEGF	heparin-binding EGF-like growth factor	−1.57	0.0044	5q23

### Gene Ontology comparing AA astrocytes and CA astrocytes

To classify differentially expressed genes, we separated the genes from RMA-SAM analysis manually by function using available data from public databases (UniGene, OMIM and Entrez PubMed). The complete list of the functional categories is shown in Table S4. In AA astrocytes compared to CA astrocytes, the most important groups of differentially expressed genes separated by function were related to signal transduction (15 genes up-regulated, 5 down-regulated), cell adhesion (4 genes up-regulated and 4 genes down-regulated), motility (3 genes up-regulated), ECM related (3 genes up-regulated and 4 genes down-regulated), responses to oxidative stress (8 genes up-regulated) and growth factors and receptors (5 genes upregulated and 2 genes downregulated) (Table 1). The number of genes that changed in selected categories is shown in Figure 1C. Classification by the Gene Ontology database (Gene Ontology Consortium) using gene list generated by Limma package yielded similar results as above (Table S5).

### Validation of selected differentially expressed genes in AA and CA astrocytes by real-time qRT-PCR

RNA from 13 AA and 17 CA age-matched normal donors was used to validate microarray data. Twenty-six genes were selected from 10 functional groups from the RMA-SAM analysis and from the GO lists. 18S was used to normalize the expression value. Fold change obtained by qRT-PCR correlated well with the direction of fold change obtained from the normalized intensity data, confirming the validity of the microarray gene expression patterns (Table S6).

### AA astrocytes exhibit altered signal transduction pathways

Genes that are associated with intracellular signaling were differentially expressed in AA astrocytes, including cAMP signaling, intracellular vesicular transport, G protein regulation and protein phosphatases (Table 1). Differences in the mRNA abundance of signaling molecules comparing AA and CA astrocytes indicate the potential for differential activation of these signaling pathways in response to stress.

#### Regulator of G protein signaling 5 (RGS5)

**Figure 2:**
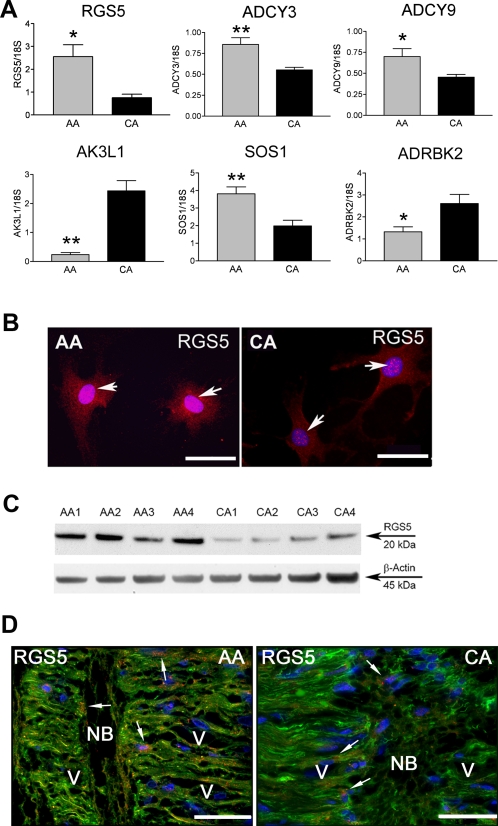
Signal transduction pathways in AA astrocytes compared to CA astrocytes. A. Confirmation of six differentially expressed signaling genes by qRT-PCR in human normal ONH astrocytes: RGS5, ADCY3, ADCY9, AK3L1, SOS1 and ADRBK2. Genes were normalized to 18S. Graphical representation of the relative mRNA levels in normal AA and CA astrocytes (n = 8, respectively, two-tailed t test was used. **indicates p<0.01 and * indicates p<0.05). B. Cellular localization of the Regulator of G protein signaling 5 (RGS5) in primary cultures of ONH astrocytes. Immunofluorecent staining of RGS5 (red) demonstrated higher levels of RGS5 protein in AA astrocytes, compared to CA astrocytes. Nuclei stained with DAPI (blue). Note that RGS5 localizes to the cytoplasm and in the nucleus of astrocytes (arrows). Magnification bar: 25 µm. C. Representative Western blots of astrocyte cell lysates with human RGS5 antibody and β-actin used as a loading control. Note that AA1-4 normal donors express more RGS5 than CA1-4 donors. D. Representative double immunofluorescent staining of RGS5 (red) astrocyte marker GFAP (green) in sections of human ONH from an AA donor (71 year-old male) and a CA donor (75 year-old male). Nuclei stained with DAPI (blue). Note strong granular staining of RGS5 in astrocytes (arrows) in the cribriform plates in the lamina cribrosa. Fewer astrocytes stain for RGS5 in the lamina cribrosa of a CA donor. V: blood vessel, NB: nerve bundle, Magnification bar 55 µm.

Expression of the RGS5 gene was upregulated in AA astrocytes by microarray and confirmed by qRT-PCR (Figure 2A). The protein product appeared greater in AA astrocytes as shown by immunostaining localized to the cytoplasm and the nucleus (Figure 2B). Western blot detected RGS5 increased protein in cell lysates of AA astrocytes (Figure 2C). Astrocytes in the lamina cribrosa tissue from normal AA donors contained abundant RGS5 compared to CA tissues (Figure 2D). The abundant expression of RGS5 in AA astrocytes suggests an inhibitory role in the regulation of signal transduction in this population.

#### Cyclic AMP signaling

Amongst genes differentially regulated in AA astrocytes were several genes that impact upon cAMP signaling. β-adrenergic receptor kinase (ADRBK2) is downregulated in AA normal astrocytes (Figure 2A). The AK3L1 gene that regulates the amount of available nucleotides in cells was also downregulated. Two adenylyl cyclases (ADYC3 and ADYC9) were upregulated in normal AA astrocytes (Figure 2A) however there were no differences in basal levels of cAMP amongst normal AA and CA astrocytes (data not shown), suggesting other components of the cAMP pathway are also involved in the regulation of cAMP basal level. Additional upregulated signaling genes were: Phosphodiesterase 4D (PDE4D) interacting protein (PDE4DIP) and SOS1, son of sevenless 1 (Table S8).

### AA astrocytes exhibit decreased cell adhesion

**Figure 3:**
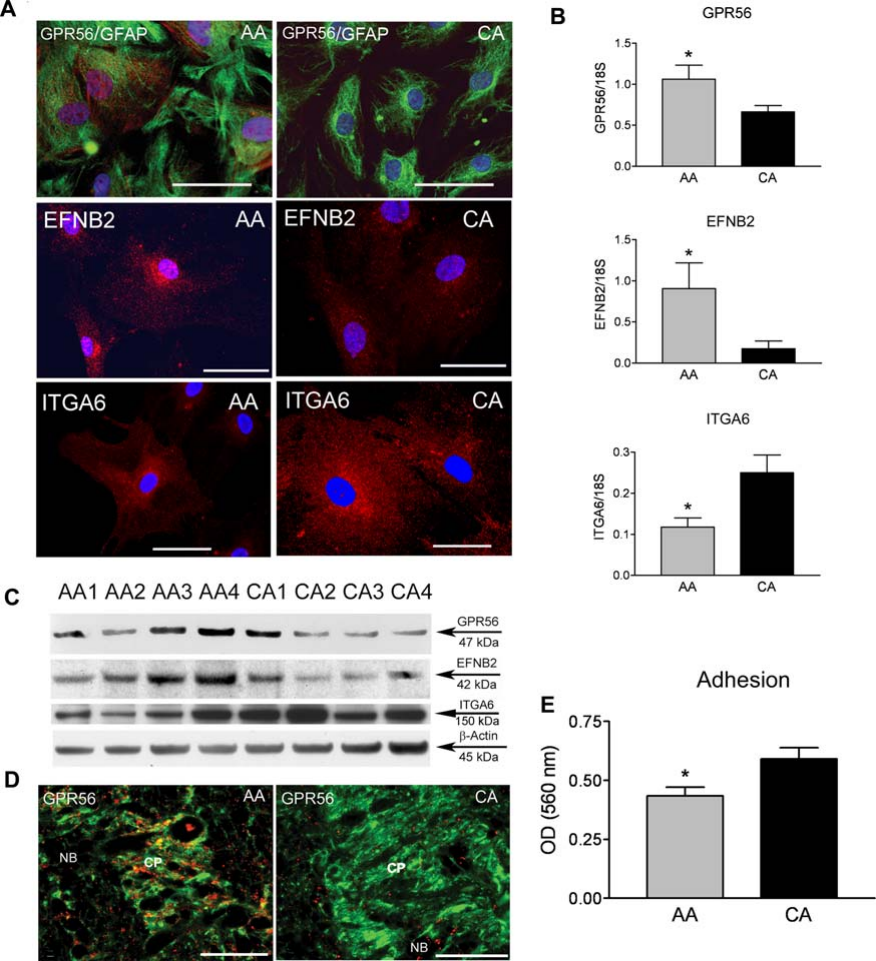
AA astrocytes exhibit decreased cell adhesion compared to CA astrocytes. A. Cellular localization of G protein-coupled receptor 56 (GPR56), ephrin-B2 (EFNB2) and integrin α 6 (ITGA6) in primary cultures of ONH astrocytes. Nuclei stained with DAPI (blue). Magnification bar: 25 µm. Upper: Double immunofluorescence for GFAP (green), an intermediate filament characteristic of astrocytes and GPR56 (red). Note granular staining for GPR56 (red) is more abundant in the cytoplasm of AA astrocytes compared to CA astrocytes. Middle: Immunofluorescence showed that EFNB2 is more abundant in the cytoplasm of AA astrocytes compared to CA astrocytes. Lower: Immunofluorescence showed that Integrin α 6 is less abundant in the cytoplasm of AA astrocytes compared to CA astrocytes. B. Confirmation of three differentially expressed adhesion genes by qRT-PCR in human normal ONH astrocytes: GPR56, EFNB2 and ITGA6. Genes were normalized to 18S. Graphical representation of the relative mRNA levels in AA and CA astrocytes (n = 8, respectively, *indicates p<0.05 in two-tailed t-test). C. Representative Western blots of astrocyte cell lysates with GPR56, EFNB2 and ITGA6 antibodies. β-actin was used as a loading control. Note that AA1-4 donors express more GPR56 and EFNB2, less ITGA6 than CA1-4 donors. D. Representative immunohistochemistry showed more abundant granular staining of GPR56 (red) in astrocytes in the lamina cribrosa from AA donors compared to CA donors. Note that GPR56 is also localized in astrocyte processes in the nerve bundles (NB). CP: cribriform plates, Magnification bar: 25 µm. E. AA astrocytes adhered to collagen IV 26.5% less than CA astrocytes did (* indicates p<0.05 in two-tailed t-test). Values represent mean optical density (OD)±standard deviation of triplicate experiments using primary astrocyte cultures of four AA donors and six CA donors.

Comparing AA to CA astrocytes, differentially expressed genes that are associated with cell adhesion were ephrin B2 and GPR56, which were both upregulated, and ITGA6, which was downregulated (Table 1, Figure 3A, and B) Differential expression in AA astrocytes was consistent with differences in the protein products of GPR56, EFNB2 and ITGA6 by immunoblot (Figure 3C). GPR56 was visibly more abundant in AA astrocytes and localized to the cell surface and cell borders *in vitro* (Figure 3A). We also detected strong staining for GPR56 in astrocytes in the lamina cribrosa of AA normal donor tissue (Figure 3D).

The molecular changes described above suggested to us decreased cell adhesion of AA astrocytes. An assay was used to compare AA astrocytes to CA astrocytes for adhesion to collagen type IV. We found decreased attachment to collagen type IV of AA astrocytes compared to CA astrocytes (Figure 3E, p<0.05).

### AA astrocytes exhibit increased migration

**Figure 4:**
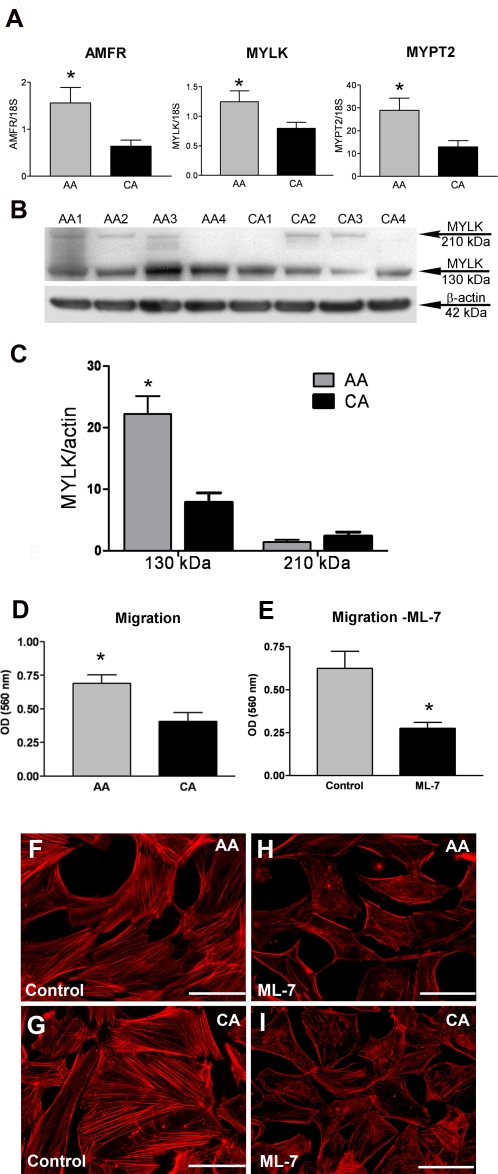
AA astrocytes exhibit increased migration compared to CA astrocytes. A. Confirmation of three differentially expressed motility genes by qRT-PCR in human normal ONH astrocytes: autocrine motility factor receptor (AMFR), myosin light chain kinase (MYLK) and myosin phosphatase target subunit 2 (MYPT2). Genes were normalized to 18S. Graphical representation of the relative mRNA levels in AA and CA astrocytes (n = 8, respectively, * indicates p<0.05 in two-tailed t-test). B. Representative Western blots of astrocyte cell lysates with MYLK antibody. β-actin was used as a loading control. Note that AA1-4 donors express more MYLK 130 kDa than CA1-4 donors. No difference was detected at the levels of 210 kDa isoforms. C. Densitometry analysis of MYLK western blots. β-actin was used as loading control. Astrocytes derived from 7 AA and 10 CA were used in this experiment. The level of the 130 kDa isoform was significantly higher in AA astrocytes, compared to CA astrocytes. D. Cell migration assay shows that AA astrocytes migrate significantly faster than CA astrocytes. The assay was performed as described in the Materials and Methods. Values represent mean optical density (OD)±standard deviation of triplicate experiments using primary astrocyte cultures of six AA donors and five CA donors. * indicate p value<0.05. E. Inhibition of MYLK by ML-7 (10 µM), leads to a decrease in migration of African American ONH astrocytes (n = 3). F, G, H, I. Phalloidin staining of the actin cytoskeleton in normal AA (F) and CA (G) astrocytes. Inhibition of MYLK by ML-7, leads to a disruption of cytoskeleton in AA (H) and CA (I) ONH astrocytes. Magnification bar: 25 µm.

mRNA levels of the autocrine motility factor receptor (AMFR), myosin light chain kinase (MYLK) a calcium/calmodulin dependent kinase and PPP1R12B (also refered as MYPT2), a myosin phosphatase were upregulated in AA astrocytes compared to CA astrocytes (Table 1, Figure 4A).

Western blots detected two isoforms of MYLK: 210 kDa and 130 kDa. The 130 kDa isoform was the predominant form in both AA and CA normal astrocytes. The protein level of MYLK 130 kDa was significantly higher in AA astrocytes compared to CA astrocytes (Figure 4B and C). 210 kDa isoform was expressed at very low levels in both AA and CA normal astrocytes and no difference was detected (Figure 4B and C).

Based on the gene expression and protein data for MYLK, we compared migration in AA and CA astrocytes. Experiments using a chemotaxis model indicated that AA astrocytes migrated faster than CA (Figure 4D). Since AA astrocytes migrated faster and have higher levels of MYLK, we tested ML-7, a known inhibitor of MYLK, on its effect on migration in astrocytes derived from three AA normal donors. The ML-7 treated cells migrated significantly slower, compare to vehicle treated cells (Figure 4E), suggesting increased migration in AA astrocytes is due at least in part to MYLK. In normal AA and CA astrocytes stained with phalloidin, stress fibers run parallel to the major axis of the cells (Figure 4F, 4G). Treatment with with ML-7, a known inhibitor of MYLK, caused a marked loss in actin stress fibers from the center of the cell (Figure 4H, 4I) supporting the role of MYLK in maintenance of the astrocyte cytoskeleton in both AA and CA astrocytes.

### Differential expression of genes associated with the extracellular matrix in AA astrocytes

**Figure 5:**
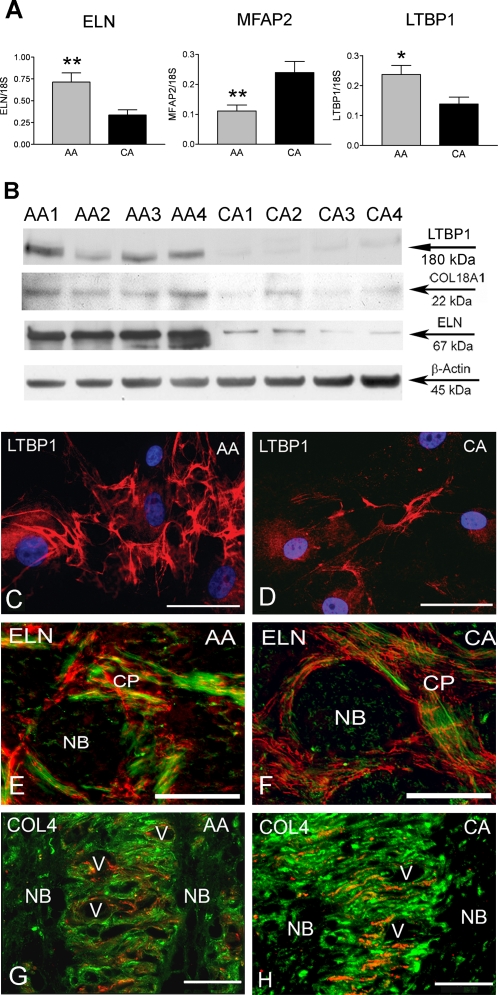
Differential expression of genes associated with the extracellular matrix (ECM) in AA astrocytes compared to CA astrocytes. A. Confirmation of three differentially expressed adhesion genes by qRT-PCR in human normal ONH astrocytes: Elastin (ELN), microfibrillar-associated protein 2 (MFAP2) and latent transforming growth factor beta binding protein 1 (LTBP1). Genes were normalized to 18S. Graphical representation of the relative mRNA levels in normal AA and CA astrocytes (n = 8, respectively, two-tailed t-test was used. ** indicates p<0.01 and * indicate p<0.05). B. Representative Western blots of astrocyte cell lysates with LTBP1, ELN and collagen type XVIII antibodies. β-actin was used as a loading control. Note that AA1-4 donors express more LTBP1, ELN and collagen type XVIII than CA1-4 donors. C, D. Immunocytochemistry of LTBP1 in AA and CA astrocytes. LTBP1 (red) is more abundant in the cytoplasm and extracellular space in AA astrocytes compared with CA astrocytes. Nuclei stained with DAPI (blue). Magnification Bar: 25 µm. E, F. Double immunostaining of ELN (red) and GFAP (green) in representative cross sectional of the ONH tissue in AA and CA donors. ELN is located in the cribriform plates and not in the nerve bundles. Astrocytes cell bodies are located in the cribriform plates (CP) and extend processes into the nerve bundle (NB). Note that there are no apparent differences in ELN staining between AA and CA samples. E is from a 75 year-old AA male donor and F is from a 74 year-old CA female donor. Colocalization between ELN and GFAP in the AA tissue is microscopic effect showing the overlap between ELN and astrocytes processes. Magnification bar: 25 µm. G, H. Double immunostaining of collagen type IV (red) and GFAP (green) in representative sagittal sections of ONH tissues from AA and CA donors. Collagen type IV and GFAP follow the lamellar structure of the astrocytic basement membranes in the human lamina cribrosa. Note that staining for collagen type IV is more intense and abundant in the CA donor than in the AA donor. G is from a 65 year-old AA male donor and H is from a 57 year-old CA male donor. V: blood vessel, Magnification bar: 35 µm.

Microarray analysis indicated a significant upregulation of elastin (ELN) mRNA which was confirmed by qRT-PCR and immunoblot in AA astrocytes (Figure 5A, B). LTBP1, a member of the elastin microfibrils that binds TGF-β was also upregulated in AA astrocytes *in vitro* (Figure 5A, B, C, and D). MFAP2, the gene encoding for MAGP1, a component of the elastin associated microfibrils, was downregulated in AA astrocytes (Figure 5A). However, ONH tissue immunostaining with ELN antibody did not show differences in ELN between populations (Figure 5 E, F). COL18A1, a collagen with strong anti-angiogenic properties, was upregulated in AA astrocytes compared to CA astrocytes by qRT-PCR and immunoblot (Figure 5B).

Several genes associated with basement membranes were downregulated in AA astrocytes (Table 1, Table S6); including Nidogen 2 (NID2), type XIII collagen [17], and PLOD2 [18]. Figures 5G and H show decreased immunostaining for collagen type IV, a ubiquitous component of basement membranes, in donor ONH tissue from AA compared to an age matched normal CA donor. AA astrocytes may be attached to an altered ECM.

### Upregulation of glutathione metabolic enzymes in AA astrocytes

**Figure 6:**
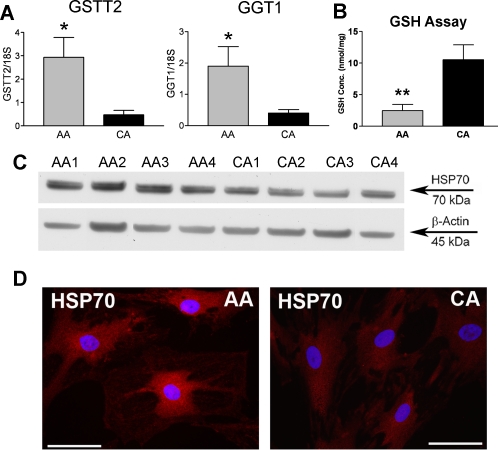
Upregulation of glutathione (GSH) metabolic enzymes in AA astrocytes. A. Confirmation of two differentially expressed glutathione metabolic enzyme genes by qRT-PCR in human normal ONH astrocytes: glutathione S-transferase theta 2 (GSTT2) and gamma-glutamyltransferase 1 (GGT1). Genes were normalized to 18S. Graphical representation of the relative mRNA levels in AA and CA astrocytes (n = 8, respectively, * indicate p<0.05 in two-tailed t-test). B. AA astrocytes have significantly lower level of intracellular GSH in vitro, compared to CA astrocytes (n = 10, ** indicated p<0.01 in two-tailed t-test). GSH content is normalized by the amount of the protein. C. Representative Western blots of astrocyte cell lysates with heat shock 70 kDa protein (HSP70) antibody. β-actin was used as a loading control. HSP70 levels are higher in AA astrocytes compared to CA astrocytes. D. Immunofluorecent staining for HSP70 shows that AA astrocytes exhibit more abundant intracellular HSP70 staining compared to CA astrocytes. Magnification bar: 25 µm.

Genes involved in GSH metabolism, including glutathione S-transferases (GSTs) and gamma-glutamyltransferases (GGTs), are upregulated in AA astrocytes (Table 1, Figure 6A). We measured basal levels of GSH in astrocytes from 10 AA lines and 10 CA lines. Consistent with upregulation of the GSH metabolizing enzymes, AA astrocytes exhibited lower GSH levels compared with CA astrocytes (Figure 6B, p<0.01).

Several chaperones were upregulated in AA astrocytes compared to CA astrocytes, including heat shock protein 70 protein 2 (HSPA2), alpha-crystallin-related heat shock protein B6 (HSPB6), and crystallin-β B2 (CRYBB2) (Table 1). HSP70 protein are more abundant in AA astrocytes compared to CA astrocytes (Figure 6C and D).

### Differential expression of growth factors and cytokines in AA astrocytes

**Figure 7:**
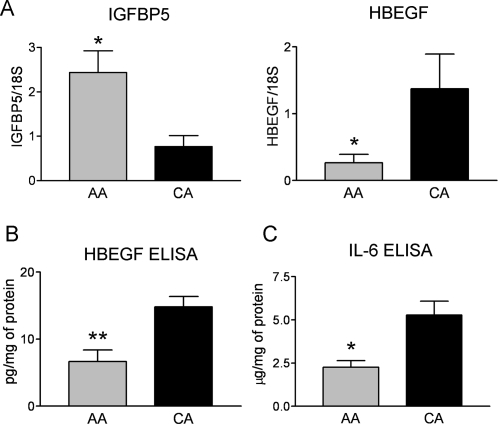
Differential expression of growth factors and cytokines in AA astrocytes. A. Confirmation of two differentially expressed growth factor genes by qRT-PCR in human normal ONH astrocytes: insulin-like growth factor binding protein 5 (IGFBP5) and heparin-binding EGF-like growth factor (HBEGF). Genes were normalized to 18S. Graphical representation of the relative mRNA levels in normal AA and CA astrocytes (n = 8, respectively, two-tailed t-test was used. * indicate p<0.05). B, C. Seven CA samples and six AA samples were used in ELISA experiment. B. The total amount of secreted and intracellular HBEGF is significantly lower in AA astrocytes compared to CA astrocytes (** indicated p<0.01 in two-tailed t-test) C. The level of secreted IL-6 is significantly lower in AA astrocytes compared to CA astrocytes (* indicated p<0.05 in two-tailed t-test).

Among the upregulated genes by microarray in AA astrocytes compared to CA astrocytes were two members of the IGFBP family, IGFBP3 and IGFBP5, the GDNF family receptor α1, the receptor for glial derived growth factor-1 and neurturin (Table 1). Real time RT- PCR confirmed increased expression of IGFBP5 (Figure 7A).

To verify the expression of growth factors and cytokines, we used ELISA to measure the quantity of both secreted (medium) and intracellular (cell lysate) forms in 6 AA and 7 CA age matched astrocyte lines. Consistent with lower mRNA levels in AA astrocytes (Figure 7A), the total amount of HBEGF was significantly lower in AA astrocytes compared to CA astrocytes (Figure 7B). IL-6 was present mainly as the secreted form in the culture medium and was significantly lower in AA astrocytes compared to CA astrocytes (Figure 7C).

## Discussion

Astrocytes are the major cell type that controls the homeostasis and microenvironment of the retinal ganglion cells axons as they traverse the optic nerve head. The complex interplay of astrocytes with RGC axons involves cell-cell signaling, synthesis of ECM, control of ions and pH, inter- and intracellular transport, immune surveillance, synthesis of growth factors and cytokines, and many other interactions. This study presents data that demonstrate population based differences (AA compared to CA) that may affect important astrocyte functions in the normal ONH. Based on data from gene expression, protein levels and functional assays *in vitro*, we hypothesize that there are *in vivo* differences in ONH astrocytes in these normal populations relating to cell adhesion/migration, intracellular signaling, extracellular matrix assembly, responses to oxidative stress, innate immune responses and neuronal survival. Population based differences in astrocyte functions may produce differential susceptibility of the ONH to elevated IOP and contribute to the pathophysiology of glaucomatous optic neuropathy in AA, in whom the disease occurs more frequently, has a younger age of onset, is more difficult to treat and progresses more rapidly compared to glaucomatous optic neuropathy in CA.

Previous work in our laboratory demonstrated that ONH astrocytes in culture retain many of the phenotypic characteristics that ONH astrocytes display *in vivo* [19], [20]. Consistent with our microarray results, differential expression of elastin, ephrin-2B, VEGF-C, PDGF-A and collagen XVIII (endostatin) has recently been demonstrated in human astrocytes in ONH tissue [20]–[22]. Immunohistochemistry indicates that many gene products detected by microarray in cultured astrocytes are present in astrocytes in the ONH *in situ*. We believe that the findings presented here using *in vitro* techniques identify functional parameters that should be investigated *in vivo*.

### Signal transduction in AA astrocytes

Amongst genes differentially regulated in AA astrocytes were several genes that impact cAMP signaling. β-adrenergic receptor kinase (ADRBK2), which is downregulated in AA normal astrocytes, specifically phosphorylates the agonist-occupied form of the β-adrenergic receptor and promotes its desensitization and internalization [23]. Thus, a decrease in ADRBK2 will likely slow β-adrenergic receptor endocytosis and prolong its activation at the membrane. In addition, two adenylate cyclases (ADCY3 and ADCY9) that are coupled to the Gs (stimulatory) GTPases activated through the β-adrenergic receptor were upregulated in AA astrocytes. Adenelyl cyclase activity is modulated by two G protein subunits: Gs to increase and Gi to decrease activity. Regulator of G protein signaling 5 (RGS5) promotes GTP hydrolysis [24] and therefore is a negative regulator of G-protein-mediated signaling. It interacts with Gi-, Go-, and Gq classes of G-proteins, but not with Gs.Therefore, the increased expression of RGS5 in AA astrocytes inhibit Gi activity, but not Gs activity. This may further enhance activation of adenylate cyclase in AA astrocytes and increase cAMP accumulation. These findings suggest increased responsiveness to β-adrenergic receptor stimulation in AA astrocytes. Similar finding has been reported for the cardiovascular system in AA [25].

### Cell adhesion/migration in AA astrocytes

AA astrocytes exhibited differential expression of genes associated with cell adhesion and cell migration. Cell adhesion genes which were downregulated in AA astrocytes included: integrin α-6 (ITGA6), which connects these cells with laminin family members [26], [27]. Basement membrane components nidogen 2 (NID2) and type XIII collagen were also downregulated in AA astrocytes.

Upregulated genes in AA astrocytes that are involved in cell adhesion of astrocytes included: GPR56, that activates pathways that inhibit cell adhesion [28] and interacts with transglutaminase 2 (TGM2), an enzyme that cross-links a various ECM proteins and ephrin B2, a cell membrane ligand that mediates repulsive forces between neighboring cells [29]. These results plus those of the adhesion assay suggest that cell-surface adhesion in the normal ONH *in vivo* by AA astrocytes may differ than that of CA astrocytes.

Cell migration involves changes in adhesion and cytoskeletal proteins and changes in cell shape during movement via reorganization of actin filament networks in the cell periphery [30]. ONH astrocytes change the distribution of the actin cytoskeleton in response to hydrostatic pressure [31]–[33].

Using microarray analysis, we have found changes in the expression of genes that suggest the machinery for migration is enhanced in AA astrocytes. In AA astrocytes, these changes included: upregulation of MYLK, a calcium/calmodulin dependent enzyme which phosphorylates myosin regulatory light chains to facilitate myosin interaction with actin filaments, producing contractile activity [34]; upregulation of AMFR, a cell surface receptor that initiates migration via activation of inositol phosphate, tyrosine kinase and protein kinase C [35] and has been characterized in glia during normal cell migration in wound healing and embryogenesis [36]; myosin phosphatase target subunit 2 (MYPT2), which dephosphorylates the phosphorylated myosin light chain to modulate the levels of contractibility in cells and is uniquely abundant in brain and in the heart [37].

MYLK genetic variants confer increased risk of sepsis and sepsis-associated with acute lung injury and a more severe asthma phenotype in individuals of African ancestry [38], [39]. Therefore it is possible that the effects of increased expression of MYLK in AA astrocytes may be further modified by genetic polymorphisms.

*In vivo*, quiescent astrocytes are terminally differentiated cells that exhibit strong and stable attachments to the ECM and neighboring astrocytes. Under conditions of stress, injury and disease, reactive astrocytes become migratory and detach from the underlying ECM. Our microarray data plus those of the migration assay obtained from primary astrocyte cultures strongly suggest that normal AA astrocytes *in situ* have the potential of becoming migratory cells in response to stress such elevated IOP as in glaucoma.

### Extracellular matrix in AA astrocytes

Our microarray data suggests that alterations in elastic fibers and associated microfibrils, a major component of the ECM in the lamina cribrosa, may be a susceptibility factor predisposing the remodeling of the ONH in response to elevated IOP in glaucoma in the AA population. Microarray analysis indicated a significant upregulation in AA astrocytes of elastin (ELN), LTBP1, a member of the elastin microfibrils that binds TGF-β, and COL18A1, a collagen with strong anti-angiogenic properties. MFAP2, the gene encoding for MAGP1, a component of the elastin associated microfibrils, and the gene encoding for LOXL2, a lysyl oxidase that participates in maturation of elastic fiber, were downregulated in AA astrocytes. We previously reported that normal AA astrocytes expressed high levels of elastin mRNA and protein, and decreased levels of LOXL2 [22]. CA patients of Scandinavian ancestry with pseudoexfoliation glaucoma exhibit variations in the LOXL1 gene sequence that may predispose this group to elevated IOP and glaucomatous optic neuropathy due to accumulation of pseudoexfoliation material [40]. We previously published marked elastosis in the ONHs of patients with pseudoexfoliation glaucoma [41]. The downregulation of LOXL2 in AA astrocytes may confer a similar susceptibility to elevated IOP.

LTBP1 is an ECM glycoprotein that plays a major role in storage of latent TGF-β in the ECM and regulate its availability [42], Most studies suggest that LTBPs are secreted together with TGF-β as part of the TGF-β large latent complex [43], [44]. LTBP1 is upregulated in AA astrocytes. The expression of ONH astrocyte-specific genes appears to be controlled by TGF-β activity [45], [46] in particular synthesis and degradation of ECM in the optic nerve in glaucoma [46]. Higher levels of TGF-β may be available and affect homeostasis of the ECM in the ONH in the AA population.

Additional ECM related genes that were downregulated included: nidogen 2 (NID2), a linker protein that joins laminin and collagen IV networks in basement membranes [47]; type XIII collagen, a type II transmembrane protein found at many sites of cell adhesion in tissues [48], and PLOD2, an enzyme that catalyzes glucosylation of type IV collagen which is essential for the formation of functional basement membranes [18]. Immunohistochemistry for collagen type IV suggested diminished basement membranes *in situ* for AA astrocytes.

Taken together, population based differences in important components of the ECM that provide elasticity and resiliency to the lamina cribrosa, and in basement membranes that participate in tissue homeostasis and adhesion of astrocytes in the ONH, may render the tissue more susceptible to the elevation of IOP that occurs in glaucoma.

### Growth factors and receptors in AA astrocytes

In normal AA astrocytes the GDNF family receptor α 1 (GDRA1) was upregulated. GDRA1 is the receptor for Glial -derived neurotrophic factor (GDNF) and neurturin (NTN) which are two potent neurotrophic factors that play key roles in the control of neuron survival and differentiation [49]. As a glycosylphosphatidylinositol (GPI)-linked cell surface receptor for both GDNF and NTN, GDRA1 mediates activation of the RET tyrosine kinase receptor and is found, with its ligands in ONH astrocytes in culture [50].

Two important members of the IGF axis, the insulin like growth factor binding protein 5 and 3 (IGFBP5 and IGFBP3) were upregulated in normal AA astrocytes. IGFBP5 interacts with heparin containing glycosaminoglycans (GAGs) in the ECM and facilitates migration, an important differentiated function of normal AA astrocytes compared to CA [51]. Among the many functions of IGFBP5 is to maintain a pool of IGF in the vicinity of cells carrying the IGF receptor thus regulating availability.

The membrane associated heparin binding EGF (HBEGF) was downregulated in AA astrocytes. Several ligands and receptors of the EGF family are known to be expressed in ONH astrocytes including HBEGF [52]. Binding of HBEGF to the EGF receptor under compressive stress is thought to be a mechanical signal that gets translated into biochemical responses by cells [33], [53]. The marked decrease in expression of HBEGF suggests a protective mechanism that normal AA astrocytes use to dampen IOP-related mechanical signals in the ONH.

### Differential expression of genes related to the immune response

AA astrocytes exhibit downregulation of expression of IL-6, a key cytokine upregulated in reactive astrocytes that promotes glial scar formation and is an impediment to axon regeneration and neuronal survival in the CNS [54]. Recent work indicated that elevated hydrostatic pressure caused a transient decrease in IL-6 levels in retinal glial cultures [55] and in human brain astrocytes exposed to hypoxia *in vitro* [56]. Decreased IL-6 in AA astrocytes may limit the transition to a reactive phenotype.

Major histocompatibility complex (MHC) Class I genes (HLA-A, HLA-B, HLA-F and HLA-G) and other molecules in the Class I antigen presentation pathway, such the immunoproteasome (PSMB9, PSMB8), were marginally up-regulated in AA astrocytes. HLA-A and HLA-B belong to the classical class I genes and are expressed in astrocytes in the CNS in disease [57]. HLA-F and HLA-G belong to the non classical class I type genes and their function is under study. Current studies of high-resolution HLA allele and haplotype frequency data are being carried out for typing and use in population-based disease studies [58]. The function of the immunoproteasome (PSMB9, PSMB8) is to process class I MHC peptides for degradation. Finally, the endoplasmic reticulum enzyme, leukocyte-derived arginine aminopeptidase (LRAP) which processes antigenic peptides presented to class I molecules was also upregulated in AA astrocytes. Expression of LRAP was reported in human brain astrocytes in response to hypoxia.

Astrocytes in the CNS participate in the innate immune response by modulating local reactions to endogenous or exogenous antigens, and by modulating astrogliosis through release of cytokines and by isolating areas of inflammation [59]. Differential expression of immune genes in AA astrocytes may have an impact on astrocyte reactivation in response to stress in glaucoma.

### Oxidative stress in normal AA ONH astrocytes

The antioxidant glutathione (GSH) is vital for cellular defense against oxidative stress in astrocytes and neurons [60]. Microarray analysis revealed upregulation of seven genes involved in GSH metabolism in AA astrocytes, including glutathione S-transferases (GSTs) and gamma-glutamyltransferases (GGTs). GSTs are a superfamily of enzymes that catalyze the conjugation of GSH to a variety of electrophilic and hydrophobic compounds. Polymorphisms in the GSTM1 and GSTT1 genes may be associated with risk of POAG [61]–[63]. GGTs initiate extracellular GSH breakdown, thus generating substrates for intracellular GSH synthesis [64] and allow a continuous ‘GSH cycling’ to occur across the plasma membrane.

Upregulation of GSTs in AA astrocytes may indicate active detoxification activity by GSH metabolizing enzymes, resulting lower levels of GSH as we demonstrated *in vitro* and perhaps *in vivo*. Upregulation of GGTs leads to increase in GSH cycling and thus GSH synthesis. The fact that GSH level is siginificantly lower in AA astrocytes, despite higher level of GGTs, suggesting there may be a compromised oxidation-reduction system or a deficient antioxidant response. Oxidative stress may be an important process in glaucomatous optic neuropathy [65]. Perhaps consistent with decreased ability to respond to oxidative stress, AA astrocytes have upregulated transcription of an array of cytoprotective genes responsible for stabilizing the cytoskeleton. In this study, several chaperones were upregulated in AA compared to CA astrocytes, including: heat shock protein 70 protein 2 (HSPA2), alpha-crystallin-related heat shock protein B6 (HSPB6), and crystallin-β B2 (CRYBB2). Upregulation of various heat shock proteins occur in the retina and ONH in response to oxidative stress and in glaucoma [65].

Our study raises many questions that will require future investigations. Our data indicates that the microenvironment supported by astrocytes in the normal ONH in AA has important differences that may impact susceptibility to glaucomatous optic neuropathy. An important question that remains is whether the observed differential gene expressions in AA astrocytes compared to CA astrocytes are the result of continued exposure to stress signals *in vivo* or represent subtle genetic changes in the AA population. Thus, differential gene expression amongst AA and CA astrocytes may provide a basis for higher risk for developing glaucoma in AA. Current studies in ONH astrocytes from glaucomatous donors further supports our hypothesis that the gene expression profile of normal AA astrocytes anticipates the changes seen in reactive astrocytes in glaucomatous optic neuropathy.

## Materials and Methods

### Human eyes

Twenty one human eyes from 21 normal age-matched Caucasian American (CA) donors (age 62±12) and 16 human eyes from 16 normal African Americans (AA) (age 60±11) were used in this study to generate primary cultures of optic nerve head (ONH) astrocytes (Table S1).

Donors did not have history of eye disease, diabetes, or chronic CNS disease. Eyes were obtained from the local eye banks and from the National Disease Research Interchange (NDRI) (Table S1). Eyes were enucleated shortly after death and maintained at 4°C. Optic nerve heads were dissected within 24 hr of death and processed to generate ONH astrocytes [14], [15]. In order to determine whether the eyes in this study did not have hidden optic nerve disease, samples of the myelinated nerves were fixed in 4% paraformaldehyde, post-fixed in osmium, embedded in epoxy resin and stained with paraphenylendiamine to detect axon degeneration [66], [67].

### Astrocytes Cultures

Cultures of human ONH astrocytes were generated as previously described [15]. Briefly, four explants from each lamina cribrosa were dissected and placed into 25-cm^2^ Primaria tissue culture flasks (Falcon, Lincoln Park, NJ). Explants were maintained in DMEM/F-12 supplemented with 10% FBS (Biowhittaker, Walkerswille, MD) and 10 µl/ml of PSFM (10,000 U/ml penicillin, 10,000 µg/ml streptomycin and 25 µg/ml amphotericin B; Gibco/BRL, Gaithersburg, MD). Cells were kept in a 37°C, 5% CO_2_ incubator. After 2–4 weeks, primary cultures were purified by using modified immunopanning procedure described by Mi and Barres (1999) [68]. Purified cells were expanded after characterization by immunostaining for astrocyte markers GFAP and NCAM as described [15]. Second passage cell cultures were stored in RPMI 1640 with 10% DMSO in liquid nitrogen until use. For each set of experiments, cells were thawed and cultured for one more passage so that sufficient cells from the same batch were available in each set of experiments.

### Oligonucleotide Microarray Analysis

Normal human eyes used for microarray were from 12 CA normal donors (age 58±11) and 12 AA normal donors (age 58±12) (Table S1). Total RNA was extracted using Qiagen RNeasy mini kits (Qiagen, Valencia, CA). RNA was then purified and quantified by measuring absorbance at 260 nm. Quality and intactness of the RNA was assessed by capillary electrophoresis analysis using an Agilent 2100 Bioanalyzer (Agilent, Palo Alto, CA). cDNA was synthesized from 2–5 µg purified RNA by using Superscript Choice system (Gibco BRL Life Technologies, Gaithersburg, MD ) and T7-(dT)24 primer (GENSET, La Jolla, CA). Using Bioassay High Yield RNA Transcript Labeling Kit (Enzo Diagnostics, Farmingdale, NY), *in vitro* transcription was carried out by using the cleaned double-stranded cDNA as a template in the presence of biotinylated UTP and CTP. Purified biotin-labeled cRNA was fragmented before the hybridization. Hybridization of the labeled cRNA to Human Genome HG U133A and HG U133A 2.0 chips (Affymetrix, Santa Clara, CA) was carried out by using Genechip Instrument System (Affymetrix) at the Genechip Core Facility of Washington University (Saint Louis, MO). The arrays were washed and stained with streptavidin-phycoerythrin (Molecular Probes, Eugene, OR) followed by scanning with an Agilent GeneArray Scanner G2500A (Agilent Technologies, Palo Alto, CA).

### Data Analysis

#### Pretreatment of Data

The first step in the analysis of the microarray data was to determine which genes were to be considered “present” or “absent.” We estimated the probe-set present/absent calls by using the Wilcoxon signed rank-based algorithm [69]. In order to reduce false positives, we removed the “absent” probe-sets from all samples.

#### Comparison between ONH astrocytes from normal AA and CA donors

We used RMA and Bioconductor in R [70] for background correction and normalization [71], [72]. Genes with significant differential expression levels were detected by SAM (significance analysis of microarray data) and ANOVA. The false discovery rate (FDR) was set ≤5%. A total of 54 chips of non-pooled RNAs represented 2 or 3 technical replicates for each of the 24 biological samples. Of the 54 chips, 18 were Human Genome U133A chips (6 biological samples) and 36 were Human Genome U133A 2.0 chips (18 biological samples). Expression values on the slightly different two Affymetrix platforms were first matched and merged in R. In addition, we also applied routines implemented in Limma of Bioconductor [73] to fit linear models to identify differentially expressed genes between both populations. A mixed effects model was used to account for the effect of technical replicates. The results are generally consistent between both methods.

#### Gene Ontology Analysis

To assess the biological significance of the gene list derived above we classified differentially expressed genes in normal AA and CA by manual separation by function using available data from public databases such as UniGene, OMIM and Entrez PubMed and the gene lists from RMA/SAM.

In addition, we used GOstats Bioconductor package [70] for the gene ontology (GO) analysis. The identification process of differentially expressed gene was similar as described in previous section. In order to get longer gene lists, the p-value threshold was set at 0.05 without FDR adjustment. The Hypergeometric test was used to identify the over-represented GO categories based on the identified gene list. Patterns of gene expression within the differentially expressed genes in each group were assessed for over-representation in the context of molecular function, biological process, and cellular component.

### Real-time quantitative RT-PCR

Independent confirmation of differential expression was conducted using 17 astrocyte cultures from CA and 13 astrocyte cultures from AA age-matched donors obtained as described earlier in Methods. Cytoplasmic RNA was isolated from cultured ONH astrocytes (passage 3) as previously described [74]. Primers sequences are described in Table S7. cDNA was synthesized using SuperScript III First-strand synthesis system (Invitrogen, CA) following manufacturer's protocol. Quantitative RT-PCR was performed by monitoring in real time the increase in fluorescence of SYBR-Green using the MyiQ Single Color Real-Time PCR Detection System (Bio-Rad; Hercules, CA). cDNA from all samples were mixed and used as standards. Serial dilutions (1∶5, 1∶10, 1∶40, 1∶160 and 1∶640) of the mixed cDNA were used for standard curves. Sample cDNA was used at 1∶20 dilution. All experiments were carried out in triplicate and astrocytes derived from one eye of each donor were used. And the relative amounts of mRNA for target genes were normalized to the amounts of reference gene RNA (18S) in each sample. The means of relative expression values were considered significantly different when p<0.05 (unpaired t- test). Primer sequence were described in Table S7.

### Western Blots

Human ONH astrocytes were grown until reaching 90% confluence and Western blot analysis was carried out as previously described [74]. Protein lysate were generated from eight AA cultures and eight CA cultures each from one eye of each individual. The antibodies names, sources and dilutions used are described in Table S8. Membranes were washed in TBS-T and then incubated with the appropriate secondary antibody conjugated to horseradish peroxidase for 1 h. For detection we used BM chemiluminescence detection system (Roche, Indianapolis, IN). Membranes were stripped and reprobed with anti-β-actin antibody as loading control. Western blots were run in duplicate, each containing four normal AA and four normal CA samples. Detail methods are described in Text S1.

### ELISA

ELISA was used to determine the content of HB-EGF and IL-6, using ELISA kits (R & D Systems) specific for each protein. Both cell lysate and culture medium were measured. When cells reached confluence, they were washed with PBS before changed into serum-free DMEM/F-12 medium. After 24 hours of incubation, medium was collected, clarified by centrifugation, and concentrated using Centriprep YM-10 (Millipore) with a molecular weight cut-off of 10,000 kDa. As a control, fresh medium was collected and concentrated in the same fashion. Cells were collected and lysed in 100 µl of 50 mM phosphate buffer with sonication. Five µl of the supernatant was used to measure protein concentration using the Pierce Protein Assay Kit (BCA method). Assay was carried out according to manufacture's protocol. The content for each sample was calculated based on the standard curve, and results were expressed as content (nmol) per mg protein. Six normal AA and seven CA samples were used. The means of the content were considered significantly different if p<0.05 (unpaired t-test).

### Cell adhesion assay

Adhesion was measured in six CA samples and four AA samples using CytoSelect™ Cell Adhesion Assay (Cell Biolabs). Cells were harvested and resuspended in serum-free DMEM/F-12 medium. For each donor, four Collagen IV coated wells and one BSA coated well were used. Fifty thousand cells were added to each well in a volume of 150 µl. For each donor, one Collagen IV well was treated with sterile serum-free DMEM F12 as a negative control. The plate was then incubated at 37°C, 5% CO_2_ for 90 minutes. The cells were then washed 4 times with 250 µl of PBS. 200 µl of cell stain solution (Cell Biolabs) was then added to each well and incubated for 10 minutes at room temperature. The stain was then removed and the cells were washed four times with 500 µl deionized water. After allowing the cells to air dry for 25 minutes, 200 µl of extraction solution (Cell Biolabs) was added to each well and placed on an orbital shaker for 10 minutes. Extracted samples' absorbance was measured at 560 nm by a Thermo Multiskan Spectrum plate reader. Results were corrected by subtracting absorbance of the negative controls. The means from AA and CA groups were considered significantly different if p<0.05 (unpaired t-test).

### Migration assay

Migratory properties of five CA samples and six AA samples were measured by CytoSelect™ 24-well cell migration assay (Cell BioLabs). Assay was performed according to the manufacture's protocol. Five hundred µl of media supplemented with 10% fetal bovine serum was added to the bottom wells. Fifty thousand cells resuspended in 300 µl serum-free medium were added to the inserts. The plate was then incubated at 37°C, 5% CO_2_ for 24 hours. After removing the media from the inserts, non-migratory cells from the interior of the inserts were removed with cotton-tipped swabs. The inserts were stained with 400 µl of cell staining solution and washed three times with water before transferred to a clean well with 200 µl of extraction solution. The absorbance of the extracted samples was measured at 560 nm by a Thermo Multiskan Spectrum plate reader. The means from AA and CA groups were considered significantly different if p<0.05 (unpaired t-test). To inhibit MYLK, 10 µM of ML-7 (EMD Biosciences) [75] was added to culture medium 24 hours before the migration assay and maintained during the assay. Assay was performed according to the manufacturer's protocol, as described above. Three AA samples were used in the assay with ML-7 inhibition. The means from treated and untreated were considered significantly different if p<0.05 (unpaired t-test).

### Glutathione (GSH) assay

Total GSH content was quantified in astrocyte cell lysates based on previously described methods using the Glutathione Assay Kit from Cayman Chemical (Ann Arbor, MI) [76]. Cells were grown to confluence in 100 mm dishes and collected in 1.0 mL sterile PBS using a disposable cell lifter. Cell suspension were centrifuged, supernatant was removed, and 100 µl of 50 mM phosphate buffer were added to the cell pellet and sonicated in icy bath for 30 min. Samples were then centrifuged for 15 min at 4°C, the supernatant was collected, and 5 µl of the supernatant were used to measure the protein concentration using the Pierce Protein Assay Kit (BCA method). The remaining supernatant was deproteinated as described in the assay protocol. Standards and samples were then aliquoted in a 96-well plate. Freshly prepared assay cocktail was added to each well, then the plate was incubated in the dark on an orbital shaker for 25 min. Absorbance was measured at 405 nm using a plate reader. Total GSH content for each sample was calculated based on the standard curve, and results were expressed as total GSH content (nmol) per mg protein. Ten AA and 10 CA normal donor samples were measured and the means of the content were considered significantly different when p<0.05 (Unpaired t-test).

### Immunohistochemistry (IHC)

Six eyes from normal Caucasian donors (CA) and six eyes from normal age-matched African American (AA) donors were used. Detail methods are described in Text S1.

### Immunocytochemistry

Primary ONH astrocytes from six normal AA donors and from six normal CA donors were used. Detail methods are described in Text S1.

## Supporting Information

Table S1 Demographic information of normal donors. Demographic information of CA and AA normal donor eyes used to generate primary cultures of ONH astrocytes.
(0.09 MB DOC)

Table S2 Genes differentially expressed in ONH astrocytes from normal AA donors compared to their caucasian counterpart using RMA SAM. List of genes differentially expressed in AA ONH astrocytes compared to CA ONH astrocytes, obtained by RMA-SAM analysis.
(0.11 MB DOC)

Table S3 Genes differentially expressed in normal AA ONH astrocytes compared to their CA counterpart using Lima. List of genes differentially expressed in AA ONH astrocytes compared to CA ONH astrocytes, obtained by using Limma package in Bioconductor.
(0.06 MB DOC)

Table S4 Functional analysis of genes differentially expressed in AA vs. CA. Functional classification of genes differentially expressed in AA ONH astrocytes compared to and CA ONH astrocytes.
(0.06 MB DOC)

Table S5 Selected Gene ontology for AA-CA comparison. Gene Ontology for genes differentially expressed in AA ONH astrocytes compared to and CA ONH astrocytes.
(0.04 MB DOC)

Table S6 Real-Time PCR validation of microarray expression analysis of normal Caucasian American and African American ONH astrocytes. Real-Time PCR validation of microarray expression analysis of normal CA and AA ONH astrocytes.
(0.08 MB DOC)

Table S7 Quantitative RT-PCR primer information. RT-PCR primer infortion
(0.10 MB DOC)

Table S8 Primary Antibodies Used in this study. Information on the primary antibodies used in this study.
(0.04 MB DOC)

Text S1 Supplemental Methods. Detailed methods for Western Blots, immunohistochemistry and immunocytochemistry.
(0.05 MB DOC)
