# Supplementary material for: Gene Expression and Functional Studies of the Optic Nerve Head Astrocyte Transcriptome from Normal African Americans and Caucasian Americans Donors
Source: PLoS One. 2008 Aug 6;3(8):e2847. doi: 10.1371/journal.pone.0002847 (PMC2518525; doi:10.1371/journal.pone.0002847)
Supplement: Table S6 — Real-Time PCR validation of microarray expression analysis of normal Caucasian American and African American ONH astrocytes. Real-Time PCR validation of microarray expression analysis of normal CA and AA ONH astrocytes. (0.08 MB DOC) [file pone.0002847.s006.doc]

**Table S6: Real-Time PCR validation of microarray expression analysis of normal Caucasian American and African American ONH astrocytes**

| **Gene** | **Description** | **CL** | **UniGene ID** | **qRT-PCR** | | **Microarray** | |
| --- | --- | --- | --- | --- | --- | --- | --- |
|  |  |  |  | **FC** | **P-value** | **FC** | **P-value** |
| ELN | elastin | 7q11.23 | Hs.252418 | 2.12 | 0.0058 | 2.2 | 0.0023 |
| GSTT2 | glutathione S-transferase theta 2 | 22q11.2|22q11.23 | Hs.1581 | 6.15 | 0.0368 | 2.82 | 0.0000 |
| GGT1 | gamma-glutamyltransferase 1 | 22q11.23 | Hs.444164 | 4.30 | 0.05 | 1.62 | 0.0004 |
| RGS5 | regulator of G-protein signalling 5 | 1q23.1 | Hs.24950 | 3.51 | 0.0211 | 2.35 | 0.0007 |
| GPR56 | G protein-coupled receptor 56 | 16q12.2-q21 | Hs.513633 | 1.60 | 0.05 | 1.62 | 0.0051 |
| PDE4DIP | phosphodiesterase 4D interacting protein | 1q12 | Hs.432315 | 3.5 | 0.006 | 2.50 | 0.0000 |
| RAB3B | Member RAS oncogene family | 1p32-p31 | Hs.123072 | 1.77 | 0.001 | 1.64 | 0.0006 |
| CA12 | carbonic anhydrase XII | 15q22 | Hs.210995 | 1.75 | 0.03 | 1.76 | 0.0075 |
| EFNB2 | ephrin-B2 | 13q33 | Hs.149239 | 5.22 | 0.05 | 1.95 | 0.0038 |
| IGFBP5 | insulin-like growth factor binding protein 5 | 2q33-q36 | Hs.369982 | 2.64 | 0.021 | 1.46 | 0.0113 |
| DDX17 | DEAD (Asp-Glu-Ala-Asp) box polypeptide 17 | 22q13.1 | Hs.528305 | 2.09 | 0.001 | 2.44 | 0.0001 |
| MSX1 | msh homeo box homolog 1 | 4p16.3-p16.1 | Hs.424414 | 5.23 | 0.03 | 2.01 | 0.0004 |
| PLA2G4C | phospholipase A2, group IVC | 19q13.3 | Hs.18858 | 2.88 | 0.03 | 1.60 | 0.0016 |
| ADCY9 | adenylate cyclase 9 | 16p13.3 | Hs.467898 | 1.53 | 0.0438 | 1.30 | 0.0300 |
| ADCY3 | adenylate cyclase 3 | 2p23.3 | Hs.391860 | 1.51 | 0.0068 | 1.27 | 0.0070 |
| SOS1 | son of sevenless homolog 1 | 2p22-p21 | Hs.278733 | 1.91 | 0.004 | 1.67 | 0.0001 |
| LTBP1 | latent transforming growth factor beta binding protein 1 | 2p22-p21 | Hs.49787 | 1.59 | 0.04 | 1.36 | 0.0243 |
| MYPT2 | protein phosphatase 1, regulatory subunit 12B | 1q32.1 | Hs.444403 | 2.24 | 0.0109 | 1.53 | 0.0026 |
| HBEGF | heparin-binding EGF-like growth factor | 5q23 | Hs.799 | -7.00 | 0.0449 | -1.57 | 0.0044 |
| MFAP2 | microfibrillar-associated protein 2 | 1p36.1-p35 | Hs.389137 | -2.15 | 0.0077 | -1.51 | 0.0016 |
| ITGA6 | integrin, alpha 6 | 2q31.1 | Hs.133397 | -2.13 | 0.015 | -1.64 | 0.0055 |
| TEK | TEK tyrosine kinase | 9p21 | Hs.89640 | -1.39 | 0.001 | -2.08 | 0.0006 |
| ADRBK2 | Adrenergic, receptor kinase 2, beta | 22q11 | Hs.517493 | -2.00 | 0.013 | -1.31 | 0.0008 |
| AK3L1 | adenylate kinase 3-like 1 | 1p31.3 | Hs.10862 | -10.27 | 0.037 | -2.38 | 0.0074 |
| MYLK | myosin, light polypeptide kinase | 3q21 | Hs.556600 | 1.57 | 0.0485 | 1.47 | 0.0435 |
| AMFR | autocrine motility factor receptor | 16q21 | Hs.295137 | 2.4 | 0.0285 | 2.79 | 0.0000 |

CL: chromosome location; FC: fold change
