# Supplementary material for: Gene Expression and Functional Studies of the Optic Nerve Head Astrocyte Transcriptome from Normal African Americans and Caucasian Americans Donors
Source: PLoS One. 2008 Aug 6;3(8):e2847. doi: 10.1371/journal.pone.0002847 (PMC2518525; doi:10.1371/journal.pone.0002847)
Supplement: Table S7 — Quantitative RT-PCR primer information. RT-PCR primer infortion (0.10 MB DOC) [file pone.0002847.s007.doc]

**Table S7: RT-PCR primer information**

| **Name** | **Accession numbers** | **Primers** | **Sequences (5' to 3')** | **Products size (bp)** |
| --- | --- | --- | --- | --- |
| 18S | x03205 | Forward | TCTAGATAACCTCGGGCCGA | 91 |
|  |  | Reverse | ACGGCGACTACCATCGAAAG |  |
| GSTT2 | NM_000854 | Forward | AGCTCGGCCATCCTGATTTAC | 102 |
|  |  | Reverse | GCCCAGGTACTCATGAACACG |  |
| GGT1 | L20493 | Forward | GAGCAGCAGCCTGTCTTGTGT | 101 |
|  |  | Reverse | GTCTCGTAGGTGTCAGCCAGCT |  |
| RGS5 | NM_003617 | Forward | AAACCAGCCAAGACCCAGAAA | 101 |
|  |  | Reverse | TTGAAACTGGCAAGTCCATAGTTGT |  |
| GPR56 | NM_005682 | Forward | CCTCCTGGTGGACTTCAGCA | 51 |
|  |  | Reverse | CTGGAATTCTTGTCCTGGAACAG |  |
| PDE4DIP | AB042557 | Forward | ACAAGCGGAACATTGAGCTGA | 91 |
|  |  | Reverse | TCAGCCCATGTTTTATCCAGATG |  |
| PLA2G4C | NM_003706 | Forward | TTAAGGAATCTGACCCTGAAAGGTT | 101 |
|  |  | Reverse | CTTTCTTGCAGCCTCAGTAATCG |  |
| RAB3B | NM_002867 | Forward | CGGACCATCACAACAGCCTATT | 101 |
|  |  | Reverse | GTAGCCCAGTCTTGGACAGCAT |  |
| EFNB2 | U16797 | Forward | CCAAATCCAGGTTCTAGCACAGA | 101 |
|  |  | Reverse | CATCCTGAAGCAATCCCTGC |  |
| CA12 | NM_001218 | Forward | CTCGCTGTCCTGGCTGTTCT | 101 |
|  |  | Reverse | GCTTCCTGGCCTTTGTACTTTACAT |  |
| MFAP2 | NM_017459 | Forward | TGCCGTGAGGAACAGTACCC | 93 |
|  |  | Reverse | GCGGAGGCTGTAGAAGCAGA |  |
| TEK | NM_000459 | Forward | GCTCCATCCAAAAGACTTTAACCA | 101 |
|  |  | Reverse | CACTGCAGACCCAAACTCCTG |  |
| ITGA6 | AV733308 | Forward | GCCAGCAAGGTGTAGCAGCTA | 101 |
|  |  | Reverse | TTGCTCTACACGAACAATCCCTTT |  |
| HBEGF | NM_001945 | Forward | TCCTGCCATTCTTCTGGTGCTACT | 113 |
|  |  | Reverse | TCAGCTCCAATGTTCCCTGTTCCT |  |
| SOS1 | NM_005633 | Forward | AAGTGGCAATGTGTGCTGACAAGG | 111 |
|  |  | Reverse | TTTGTTCTCCTGAGGTGGAAGGCT |  |
| ADCY3 | AF033861 | Forward | TTGACTGCTACGTGGTGGTCATGT | 114 |
|  |  | Reverse | TGCAGAGCACGAAGAGGATGATGT |  |
| ADCY9 | AF036927 | Forward | TCTCCTGCTCTTGTTGGTCTGGTT | 147 |
|  |  | Reverse | ATGATGTTCCTCAGCAGCCAGTCT |  |
| AMFR | AF124145 | Forward | AACTCTGGTGCTCCTGCATCTCAT | 110 |
|  |  | Reverse | AACCAAAGCTTGCTGCCTAACCAC |  |
| ELN | NM_000501 | Forward | CCCCAAGCTGCCTGGTG | 101 |
|  |  | Reverse | AACCAGCCTTGCCCGC |  |
| MYLK | NM_005965 | Forward | TAGCATCATGAACTGCCTCCACCA | 129 |
|  |  | Reverse | TCGTCAATGATGCGCTCAAACAGC |  |
| AK3L1 | NM_001005353 | Forward | TGCTGCCAGGCTAAGACAGTACAA | 199 |
|  |  | Reverse | TCTTCCTGGTTCTTCCATTGGGCA |  |
| ADRBK2 | NM_005160 | Forward | CTGGAACACGTGCACAATCGGTTT | 110 |
|  |  | Reverse | ATCGCAGGCAAGACCAAGATCTGA |  |
| PPP1R12B | NM_002481 | Forward | GCAATTTGTTGCAGGCAGCCTTTG | 92 |
|  |  | Reverse | AAGCTGAGGAAAGGTGGTTGAGGA |  |
| IGFBP5 | NM_000599 | Forward | CGAGCAAGTCAAGATCGAGAGAG | 104 |
|  |  | Reverse | TGTGTTTGGGCCGGAAGAT |  |
| DDX17 | NM_006386 | Forward | AGGATGCGCAGAGATGGTTG | 101 |
|  |  | Reverse | GGTGCCTTTCCAGAACGGA |  |
| MSX1 | NM_002448 | Forward | CGAAGTCTGATCCCTGCCAA | 96 |
|  |  | Reverse | TCAGTTTCCCCATCTTTAACTCGA |  |
| LTBP1 | AI986120 | Forward | CATACTCCCACCAGCAGGTCAT | 101 |
|  |  | Reverse | TTTGCCACACTGTGACCCAAT |  |
