# Supplementary material for: Gene Expression and Functional Studies of the Optic Nerve Head Astrocyte Transcriptome from Normal African Americans and Caucasian Americans Donors
Source: PLoS One. 2008 Aug 6;3(8):e2847. doi: 10.1371/journal.pone.0002847 (PMC2518525; doi:10.1371/journal.pone.0002847)
Supplement: Table S8 — Primary Antibodies Used in this study. Information on the primary antibodies used in this study. (0.04 MB DOC) [file pone.0002847.s008.doc]

**Table S8: Primary Antibodies Used in this study**

| **Antibody Name** | **Species** | **Manufacturer** | **Dilution WB** | **Dilution IHC** |
| --- | --- | --- | --- | --- |
| regulator of G protein signaling 5 (RGS5) | Rabbit polyclonal | Imagenex | 1:1000 | 1:100 |
| G protein-coupled receptor 56 (GPR56) | Rabbit polyclonal | Novus Biologicals | 1:2000 | 1:100 |
| Ephrin B2 (EFNB2) | Rabbit polyclonal | Santa Cruz Biotechnology | 1:1000 | 1:100 |
| collagen type XVIII | Mouse monoclonal | Upstate biotechnology | 1:500 | 1:100 |
| Elastin (ELN) | Rabbit polyclonal | Elastin Products Company | 1:1000 | 1:100 |
| myosin light chain kinase (MYLK) | Mouse monoclonal | Sigma | 1:10000 | 1:500 |
| heat shock 70kDa protein (HSP70) | Mouse monoclonal | Santa Cruz Biotechnology | 1:1000 | 1:50 |
| latent transforming growth factor beta binding protein 1 (LTBP1) | Mouse monoclonal | R&D system | 1:1000 | 1:200 |
| β-actin | Mouse monoclonal | Sigma | 1:10000 | Not used |
| integrin α 6 (ITGA6) | Mouse monoclonal | Santa Cruz Biotechnology | Not used | 1:100 |
| collagen type IV | Rabbit polyclonal | Chemicon | Not used | 1:50 |

WB: Western blot; IHC: immunohistochemistry.
