# Supplementary material for: Gene Expression and Functional Studies of the Optic Nerve Head Astrocyte Transcriptome from Normal African Americans and Caucasian Americans Donors
Source: PLoS One. 2008 Aug 6;3(8):e2847. doi: 10.1371/journal.pone.0002847 (PMC2518525; doi:10.1371/journal.pone.0002847)
Supplement: Text S1 — Supplemental Methods. Detailed methods for Western Blots, immunohistochemistry and immunocytochemistry. (0.05 MB DOC) [file pone.0002847.s009.doc]

**Text S1: Methods**

***Western Blot***

Human ONH astrocytes were grown in 6-well plates until reaching 90% confluence. Cells were then washed twice with cold PBS (Dulbecco’s Phosphate buffered Saline, Invitrogen Corporation) before lysed with 200-400 μl of ice-cold immunoprecipitation (IP) buffer containing 10 mM Tris–HCl, pH 7.5, 150 mM NaCl, 1 mM EDTA, 0.05% Triton X-100 and Roche protease inhibitors Complete Mini ^TM^ (Roche Molecular Biochemicals; Indianapolis, IN). After incubation in IP buffer for 15 min, cells were scraped with disposable cell lifters and transferred to Eppendorf tubes, and centrifuged for 15 min at 4 °C and 14,000 rpm. The supernatant was transferred into fresh pre-cooled microcentrifuge tubes. The protein concentrations were determined by BCA protein assay kit (Pierce, Rockford, IL). Samples (3-15 μg per lane) were run on NuPAGE 4–12% Bis-Tris Gel and transferred to Hybond-P PVDF membranes. Membranes were blocked for 30 min in blocking solution [0.5% BM Chemiluminescence blocking reagent (Roche, Indianapolis, IN) in Tris buffered saline solution (TBS)] and incubated overnight with primary antibody diluted in TBS/0.5% blocking reagent. Primary antibody information is listed in Table S8. Membranes were washed in TBS-T and then incubated with the appropriate secondary antibody conjugated to horseradish peroxidase for 1 h at room temperature. Detection occurred with BM chemiluminescence detection solution (Roche, Indianapolis, IN). Membranes were stripped and reprobed with anti-β-actin antibody (1:10000: Sigma) as loading control. Protein lysate samples were generated from eight AA cultures and eight CA cultures. Western blots were run in duplicate, each containing four normal AA and four normal CA samples.

***Immunohistochemistry:***

Six eyes from normal Caucasian donors (CA) and six eyes from normal African American (AA) donors were used. Tissues were fixed with 4% paraformaldehyde in 0.1 M phosphate-buffered saline (PBS) pH 7.4 and processed for paraffin embedding. Two slides were stained per donor containing at least two six µm optic nerve sections each. A list of primary antibodies is included as Table S8. Double immunohistochemistry used monoclonal and polyclonal antibodies against human glial acidic fibrillar protein (GFAP) as an astrocyte marker. We used secondary antibodies labeled with Alexa 488 and Alexa 568 (1:800; Molecular Probes, Eugene, OR). For negative controls, the primary antibody was replaced with non-immune serum. Serial sections of normal AA and CA eyes were stained simultaneously to control for variations in immunostaining. Slides were examined in a Nikon Eclipse 80*i* microscope (Tokyo, Japan) equipped with epifluorescent illumination and digital cameras (CoolSnap ES and CF, Photometrics). The images were processed using Meta Imaging Series software (Molecular Devices) and stored as a computer files.

***Immunocytochemistry***

Cells from six normal AA donors and from six normal CA donors were used. Astrocytes grown on coverslips were fixed in 4% paraformaldehyde in phosphate-buffered saline (PBS) and processed for standard indirect immunofluorescence. Fixed coverslips were washed in PBS with 0.5% bovine serum albumin (BSA) (0.5% BSA/PBS) and permeabilized with 0.1% Triton X-100 in distilled water. Coverslips were blocked with 10% donkey serum (Sigma) in 0.5% BSA/PBS for 30 min. Coverslips were processed for single immunofluorescence staining using antibodies listed on Table S8 or for double immunofluorescence using monoclonal GFAP antibody and the appropriate primary antibody. Each first antibody was applied for 2 h at room temperature followed blocking serum and by incubation with the second primary antibody for 2 h at room temperature. After each primary antibody, followed by washes with PBS, coverslips were incubated with Alexa Fluor 488 goat anti-mouse IgG and Alexa Fluor 568 goat anti-rabbit IgG (Molecular Probes, Eugene, OR) secondary antibodies diluted in 0.5% BSA/PBS. Controls were incubated with non-immune serum. To detect the actin cytoskeleton, astrocytes were stained with Texas-Red-labeled phalloidin (1:40; Molecular Probes). After washing with 0.5% BSA/PBS, cells were rinsed with PBS and mounted on slides using Vectashield mounting medium with or without DAPI (Vector Laboratories, Burlingame, CA). Coverslips were examined in a Nikon Eclipse 80*i* microscope (Tokyo, Japan) equipped with epifluorescent illumination and digital cameras (CoolSnap ES and CF, Photometrics). The images were processed using Meta Imaging Series software (Molecular Devices) and stored as a computer files.
